# Anterior staphyloma of right eye: a rare clinical image

**DOI:** 10.11604/pamj.2022.42.189.35689

**Published:** 2022-07-07

**Authors:** Tejaswee Lohakare, Khushbu Meshram

**Affiliations:** 1 Department of Child Health Nursing, Smt Radhikabai Meghe Memorial College of Nursing, Datta Meghe Institute of Medical Sciences, Sawangi (Meghe), Wardha, Maharashtra, India

**Keywords:** Anterior staphyloma, ectasia, uvea, right eye, loss of vision

## Image in medicine

Staphyloma is a thinning (ectasia) of the globe's uvea and scleral layers. It causes globe expansion with a focal bulge, commonly in the posterior wall. Anterior staphyloma can form as a consequence of an inflammatory/infectious event or as a result of a developmental abnormality caused by defective anterior mesoderm differentiation. The congenital form is uncommon. We present a case of an 8-year-old female child who was brought to the outpatient department with a complaint of pain and loss of vision from the right eye. As narrated by her parents she was alright 2 years, back then she had a trauma on her right eye while playing; after which she started developing ulceration over the right eye and it kept increasing in size. After physical examination of the eye, discharge was present from the right eye, vision loss and redness in eyes. The patient was referred to the ophthalmologist for further examination and investigation, where an anterior staphyloma of the right eye was diagnosed. The patient was referred to the ophthalmology department for further management.

**Figure 1:**
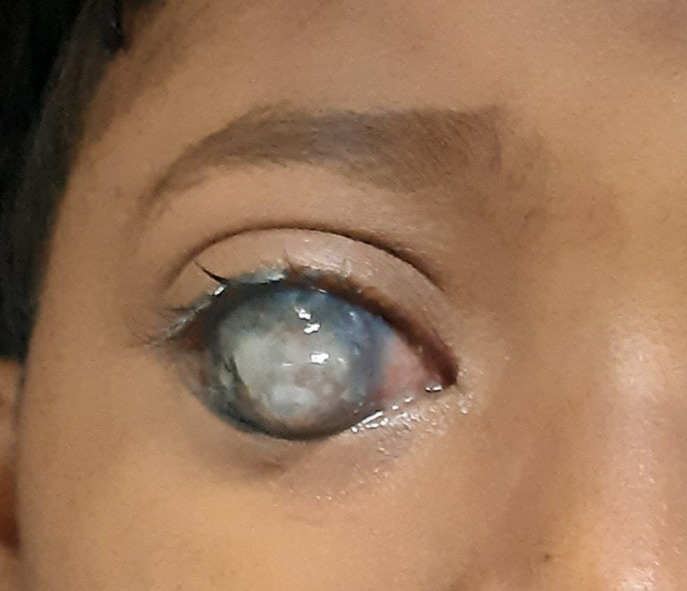
ulceration over anterior side of right eye

